# Ultra-thin clay layers facilitate seismic slip in carbonate faults

**DOI:** 10.1038/s41598-017-00717-4

**Published:** 2017-04-06

**Authors:** Luca Smeraglia, Andrea Billi, Eugenio Carminati, Andrea Cavallo, Giulio Di Toro, Elena Spagnuolo, Federico Zorzi

**Affiliations:** 1 Dipartimento di Scienze della Terra, Sapienza University of Rome, Rome, Italy; 2 Consiglio delle Nazionale Ricerche, IGAG, Rome, Italy; 3 CERTEMA, Multidisciplinary technology laboratory, Cinigiano, Grosseto Italy; 4 School of Earth, Atmospheric and Environmental Sciences, The University of Manchester, Manchester, UK; 5 INGV, Istituto Nazionale di Geofisica e Vulcanologia, Rome, Italy; 6 Dipartimento di Geoscienze, Padova University, Padova, Italy

## Abstract

Many earthquakes propagate up to the Earth’s surface producing surface ruptures. Seismic slip propagation is facilitated by along-fault low dynamic frictional resistance, which is controlled by a number of physico-chemical lubrication mechanisms. In particular, rotary shear experiments conducted at seismic slip rates (1 ms^−1^) show that phyllosilicates can facilitate co-seismic slip along faults during earthquakes. This evidence is crucial for hazard assessment along oceanic subduction zones, where pelagic clays participate in seismic slip propagation. Conversely, the reason why, in continental domains, co-seismic slip along faults can propagate up to the Earth’s surface is still poorly understood. We document the occurrence of micrometer-thick phyllosilicate-bearing layers along a carbonate-hosted seismogenic extensional fault in the central Apennines, Italy. Using friction experiments, we demonstrate that, at seismic slip rates (1 ms^−1^), similar calcite gouges with pre-existing phyllosilicate-bearing (clay content ≤3 wt.%) micro-layers weaken faster than calcite gouges or mixed calcite-phyllosilicate gouges. We thus propose that, within calcite gouge, ultra-low clay content (≤3 wt.%) localized along micrometer-thick layers can facilitate seismic slip propagation during earthquakes in continental domains, possibly enhancing surface displacement.

## Introduction

Many earthquakes propagate up to the Earth’s surface producing surface ruptures, possibly associated with tsunami generation, infrastructure damage, and fatalities^1–4^. Seismic slip propagation is facilitated by low dynamic frictional resistance along faults^5–7^. Therefore, understanding the mechanisms that lower rock resistance and facilitate seismic slip propagation along faults^8^ is relevant for hazard assessment within seismically active regions. To understand such a mechanism, rotary shear experiments simulating natural seismic deformation conditions have been conducted on different natural fault rocks at seismic slip rates (≥1 ms^−1^) in the past 20 years^5, 9^. Results show that a number of processes triggered by high mechanical work rates can induce fault weakening^10, 11^, especially within thermally-unstable rocks such as carbonates (i.e., thermochemical pressurization and grain-size dependent processes^11–18^). In particular, experiments have revealed the weakening behavior of phyllosilicates, especially in water-saturated conditions, due to their low steady-state dynamic friction and to the absence of peak friction^19–23^. For this reason, previous studies^24–27^ suggested that pelagic clay commonly present along offshore subduction zones participate to co-seismic fault lubrication, possibly facilitating and enhancing upward co-seismic slip propagation, sometimes associated with tsunamigenic seafloor displacement. Less understood is the origin of surface displacements associated with moderate (M_w_ ≤ 7.0) earthquakes in continental seismically-active regions^4, 28, 29^ (see Supplementary Table S1), as in such settings clay occurrence can be very modest. Here we propose that less than 3 wt.% of phyllosilicates localized within pre-existing ultra-thin layers along carbonate-hosted faults can facilitate co-seismic slip propagation, possibly enhancing surface displacement. In particular, we first document the occurrence in nature of hitherto unknown phyllosilicate-bearing micrometer-thick layers along the carbonate-hosted seismically-active Tre Monti Fault^30–32^ and then we use a rotary shear apparatus to simulate the behavior of this natural setting during co-seismic slip propagation.

**Figure 1:**
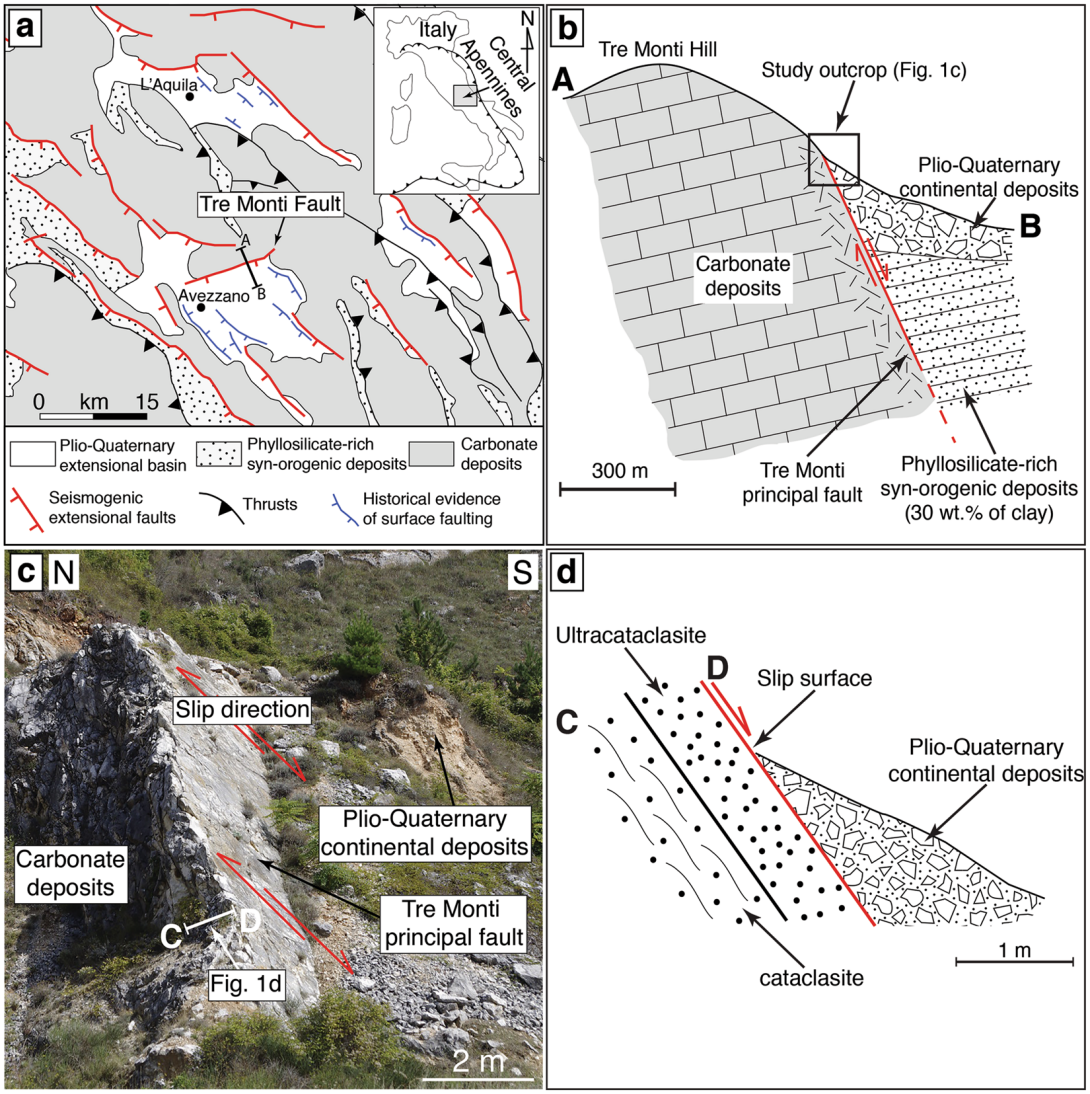
Geological setting. (**a**) Geological setting of the Tre Monti Fault and evidence of surface faulting from historical earthquakes in the central Apennines, Italy. Geological map and base map has been drawn using Adobe Illustrator CS5. **(b**) Simplified geological cross-section through the Tre Monti Fault. Cross-section trace in (**a**). Cross-section has been drawn using Adobe Illustrator CS5. **(c)** The study outcrop of the Tre Monti principal fault (Latitude 42°04′34″ N, Longitude 13°29′58″ E). **(d)** Simplified structural cross-section through the Tre Monti principal fault showing the ultracataclasite above the foliated cataclasite. Cross-section has been drawn using Adobe Illustrator CS5.

The Tre Monti Fault is a shallow (exhumed from depths <3 km^31^) seismogenic extensional fault belonging to the carbonate domain of the central Apennines, Italy^30–33^ (Fig. 1a). The central Apennines is a fold-thrust belt (Oligocene-Quaternary) characterized by imbricate thrust sheets consisting of ~4–5 km thick pre-orogenic massive platform carbonates overlain by ~2 km thick phyllosilicate-rich syn-orogenic deposits (i.e., hemipelagic marls and foredeep clayey-sandstones)^34–36^. Massive carbonate rocks are the main lithology of the central Apennines down to at least 7–8 km depth^37, 38^. During post-orogenic extension (Pliocene to present time), the fold-thrust belt has been dissected by a system of extensional faults, which bound several intramountain basins filled by Plio-Quaternary continental deposits^39, 40^. These faults have generated historical and instrumental seismicity up to M_w_ 7.0^41^ (Fig. 1a; e.g., Avezzano, 1915, M_w_ 7.0 earthquake; L’Aquila, 2009, M_w_ 6.1 earthquake; Amatrice-Norcia, 2016, M_w_ 6.0 and 6.5 earthquakes). In this region, earthquakes mainly nucleate at depths ≤8 km and co-seismic slip propagates upward along carbonate-hosted faults, often generating surface displacement^4, 30, 42, 43^ (Fig. 1a), as also observed in other seismically-active areas worldwide (see Supplementary Table S1). Therefore, geological and mechanical studies of faults exhumed from shallow depths, such as the Tre Monti Fault, are significant as the small-scale compositional heterogeneity of shallow fault zones can promote or inhibit seismic slip propagation up to the Earth’s surface.

## Results

### Natural fault zone structures

**Figure 2:**
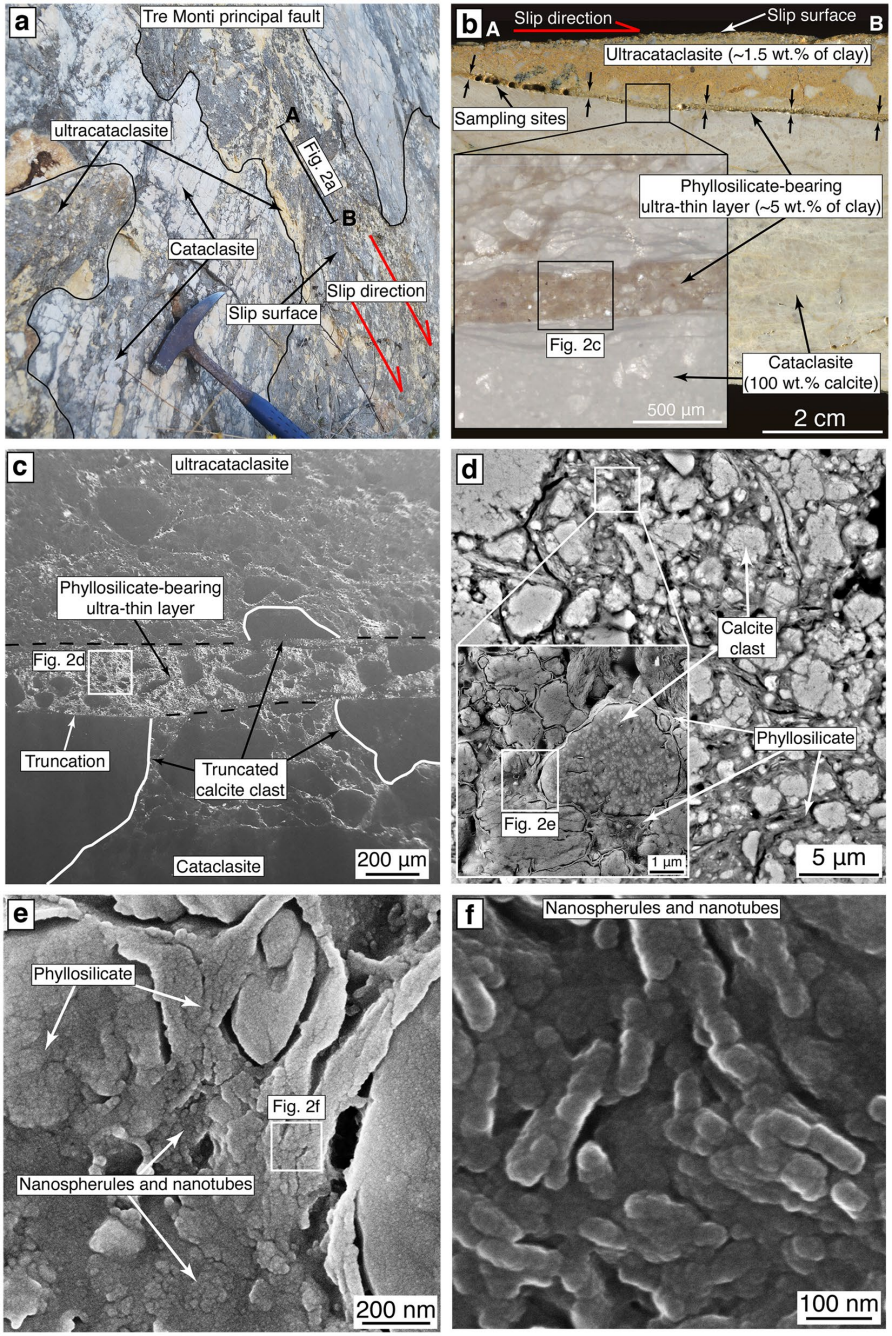
Phyllosilicate occurrence within the carbonate-hosted Tre Monti Fault. **(a)** Detail of the Tre Monti principal fault showing patches of ultracataclasite above the foliated cataclasite. **(b)** Phyllosilicate-bearing layer at the boundary between the ultracataclasite and the foliated cataclasite. Inset shows a microphotograph under optical microscope showing a detail of the phyllosilicate-bearing layer. **(c)** FE-SEM image of micrometer-thick phyllosilicate-bearing layer, which truncates calcite clasts. **(d)** FE-SEM image of carbonate clasts within a phyllosilicate matrix. Inset shows calcite clasts wrapped by a continuous phyllosilicates film. **(e–f)** FE-SEM images of nanostructures of natural phyllosilicates from the phyllosilicate-bearing layers characterized by clumped and chained nanospherules and nanotubes.

The Tre Monti Fault juxtaposes pre-orogenic carbonate deposits (footwall) with both syn-orogenic phyllosilicate-rich deposits (Fig. 1b; average phyllosilicate content ~30%, see Supplementary Table S2) and overlying continental (fluvial, lacustrine, and slope) deposits (hangingwall). The fault displays a dip of ~60° and a maximum displacement of ~1,500–2,000 m^31, 44^. The principal fault surface is excellently exposed along a ~8 km long carbonate-hosted fault scarp^30–32^ (Fig. 1c) exhumed by multiple surface faulting events^26^. The principal fault is associated with a ~1 m-thick cataclastic fault core, which consists of an ultracataclasite lying above a cataclasite^27^ (Figs 1d and 2a–c). The ultracataclasite shows more intense grain comminution than the cataclasite (Fig. 2c) and forms discontinuous patches and plagues above the cataclasite owing to fault scarp erosion. X-ray Powder diffraction (XRPD) and energy dispersive spectroscopy (EDS) analyses revealed that the cataclasite consists solely of calcite, whereas the ultracataclasite consists of calcite, phyllosilicates (~1.5 wt.%; mainly smectite, illite, and kaolinite; see Supplementary Fig. S1 and Supplementary Table S2), detrital micas, quartz, K-feldspar, and plagioclase (<1%; see Supplementary Fig. S2 and Supplementary Table S3), which are also present within the syn-orogenic deposits (see Supplementary Table S2)^45, 46^.

Phyllosilicates are concentrated within slip-parallel micrometer-thick (from ~30 μm up to ~1 mm in thickness) layers at the boundary between the ultracataclasite and the cataclasite (Fig. 2b and c). The average phyllosilicate content within these layers is ~5 wt.% (see Supplementary Fig. S2 and Table S2). Phyllosilicate-bearing layers have sharp boundaries, which truncate calcite clasts (Fig. 2b and c). Within these layers, calcite clasts are scattered within a phyllosilicate matrix and are usually wrapped by phyllosilicates creating a continuous film (Fig. 2d). At the nanoscale, phyllosilicates consist of granular zones and elongated fibers comprising clumped and chained ~50-nm-thick nanospherules and nanotubes (Fig. 2e and f).

Due to lack of evident dissolution seams and teeth-shaped margins (i.e., typical of stylolites^47, 48^) close to the phyllosilicate-bearing layers, we exclude that these layers formed by residual processes connected with pressure-solution of carbonates. Moreover, XRPD and EDS analyses show that the non-carbonate minerals of the ultracataclasite (Supplementary Tables S2 and S3) are identical to those of syn-orogenic deposits in the fault hangingwall (Supplementary Table S2). Hence, we infer that the phyllosilicates derived from the phyllosilicate-rich syn-orogenic deposits at the fault hangingwall, and that they were smeared and mixed into the ultracataclasite during fault activity^49^. Although we cannot totally exclude phyllosilicates derived by pressure-solution of carbonates, we suggest that the contribution of phyllosilicates derived by such processes (if any) can be neglectable^49^. However, independently from the mode of phyllosilicate concentration along thin layers (i.e., through clay smearing, pressure-solution of carbonates, and so on) in the TMF or in another carbonate-hosted faults, in the following section we emphasize the mechanical role of pre-existing phyllosilicate-bearing layers during co-seismic slip propagation.

### Laboratory experiments and comparison with natural fault products

Based on the previously-reported field, mineralogical, and microscopic observations, we conducted low to high velocity rotary shear friction experiments (see Methods) to investigate the frictional behavior of micrometer-thick phyllosilicate-bearing layers within synthetic calcite gouges reproducing those observed along the Tre Monti Fault (Fig. 2b and c). We performed experiments on 3 mm-thick gouge layers obtained by crushing and sieving (<125 µm) the natural foliated cataclasite (100% calcite) and the phyllosilicate-rich syn-orogenic deposits (~30% of phyllosilicates), both under dry and under water-saturated (wet) conditions.

**Figure 3:**
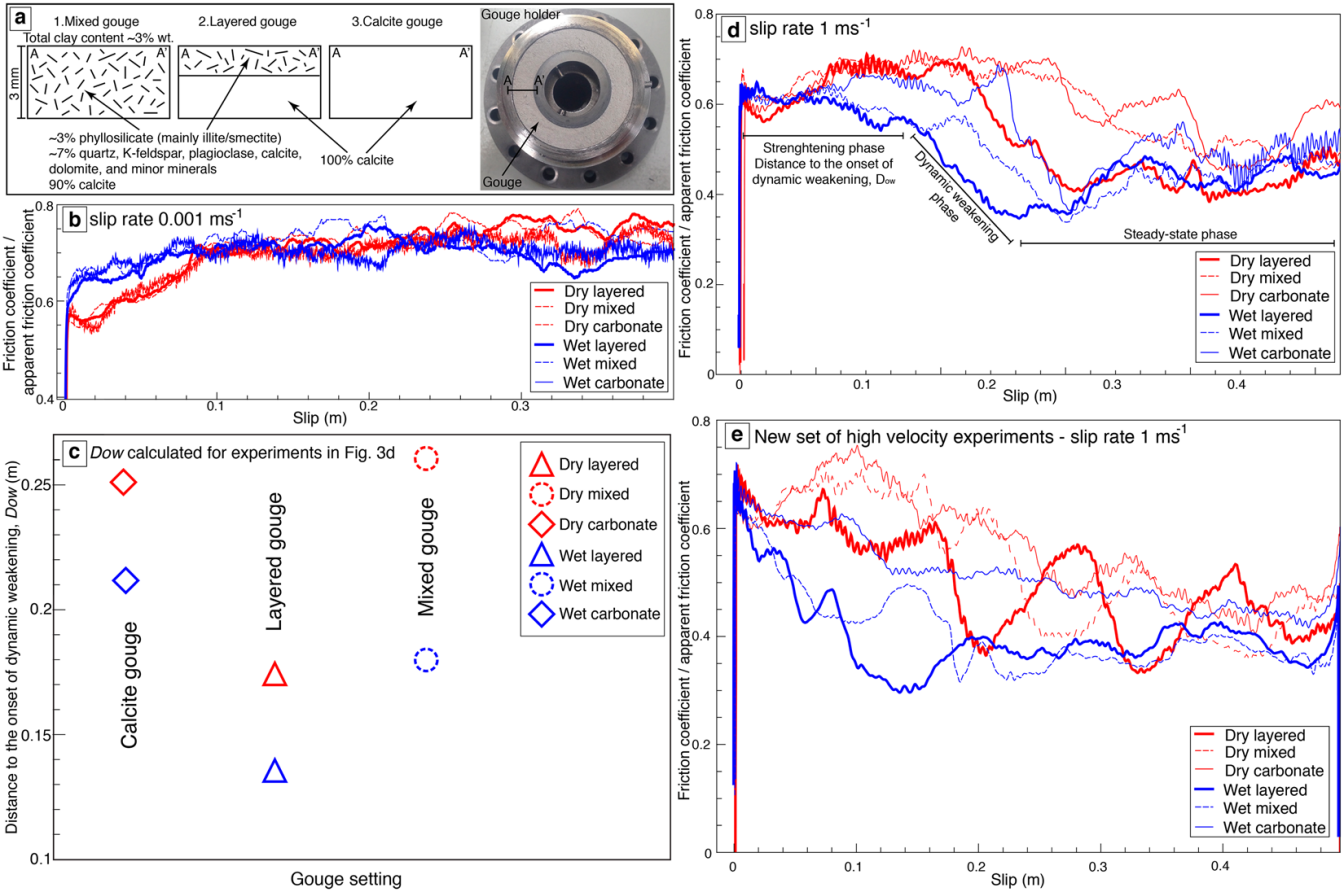
Results from friction experiments. **(a)** experimental setup: (left) the three different configurations of tested gouges and (right) the gouge holder. (**b**) Friction coefficient versus slip during experiments at subseismic (0.001 ms^−1^) slip rate. **(c)** Distance to the onset of dynamic weakening, *D*_*ow*_, versus clay content. Layered gouges are characterized by a *D*_*ow*_ lower than those of mixed and calcite gouges, both in dry and in wet conditions. **(d,e**) Friction coefficient versus slip during experiments at seismic (1 ms^−1^) slip rate.

Experimental slip rates were set at 0.001 ms^−1^ and 1 ms^−1^ to simulate subseismic and co-seismic slip rates, respectively. We held the normal stress constant at 8.5 MPa and 5 MPa in dry and wet experiments, respectively, consistently with lithostatic stress at shallow depth (<0.5 km). The SHIVA apparatus does not allow the measurement/control of pore pressure during the wet experiments. Accordingly the measured friction should be considered as apparent (or effective), as suggested by Chen *et al*. (2017), for the unknown pore pressure within the gouge. We set normal stress to 5 MPa under wet conditions to avoid gouge extrusion from the gouge holder. In all experiments, gouges were initially pre-sheared at 0.01 ms^−1^ for 10 cm of total slip under 0.5 MPa of normal stress. This procedure was done to properly simulate the behaviour of our natural fault rocks (Fig. 2b), which were (pre-) sheared by multiple slip events and to limit the pressurization effects due to gouge compaction, as also done in previous experiments in rotary shear apparatuses^50^. We sheared the gouges imposing a total slip of 0.5 meter, both during low- (0.001 ms^−1^) and high-velocity (1 ms^−1^) experiments, to simulate, in the latter case, the amount of co-seismic slip occurring at depth <0.5 km during a M_w_ 6.0–6.5 earthquake^51^. We carried out experiments using three different gouge configurations (Fig. 3a) aimed at understanding the mechanical effect of a small percentage of clay concentrated along ultra-thin layers within the calcite gouge. The configurations are: (1) 10% of phyllosilicate-rich deposits randomly mixed with 90% of calcite (“mixed gouge experiments”; total phyllosilicate content of ~3 wt.%); (2) 2 mm-thick layer of 100% calcite overlain by a 1 mm-thick layer consisting of a random mixture of 10% of phyllosilicate-rich deposits and 90% of calcite (“layered gouge experiments”; total phyllosilicate content in the 1-mm thick layer of ~3 wt.%); this configuration, in particular, simulates the phyllosilicate-bearing layers observed within natural samples from the Tre Monti Fault (Fig. 2b and c). For this configuration, we also performed a non-shear compression experiment to study the pre-shear microstructures (compare Supplementary Fig. S3 with Fig. 3a “layered gouge” configuration); (3) 100% calcite gouge (“calcite gouge experiments”). Complete experimental procedures and results are listed in the method section and Supplementary Table S4.

At subseismic slip rates (0.001 ms^−1^), all gouges have a similar friction/apparent friction coefficient evolution from the first peak value to a steady-state value (0.7–0.8; Fig. 3b). At seismic slip rates (1 ms^−1^), all experiments are characterized by a dynamic weakening behavior. In particular, during the decay of friction/apparent friction (i.e., the weakening phase) towards a steady-state value (~0.4) occurs after a strengthening phase defined as the distance (i.e., slip) to the onset of dynamic weakening (*D*_*ow*_^52^; Fig. 3c and d). We calculated the *D*_*ow*_ values considering the maximum distance reached by the strengthening phase until the start of the weakening phase (Fig. 3c and d). Remarkably, we recognize a general trend showing that layered gouges weaken faster and have lower fracture energy than mixed gouges, which, in turn, weaken faster and have lower fracture energy than carbonate gouges both in dry and in wet conditions (Fig. 3c and d, and Supplementary Table S4). In particular, layered gouges have a *D*_*ow*_ shorter than those measured for mixed and calcite gouges, both in dry and in wet conditions (Fig. 3c and d). Overall, wet gouges show a lower steady-state dynamic friction values than dry gouges, as previously observed within water-saturated gouges^20–22^ (Fig. 3d).

To verify the reproducibility of the mechanical data, we replicated a set of six additional experiments both in wet (three experiments: mixed, layered, and calcite gouge experiments; Fig. 3e) and in dry conditions (three experiments: mixed, layered, and calcite gouge experiments; Fig. 3e). Despite the high heterogeneity of the tested materials (i.e., natural rocks such as syn-orogenic deposits), these experiments reproduce well the previously observed mechanical trend: layered gouges weaken faster than mixed gouges, which, in turn, weaken faster than calcite gouges both in wet and dry conditions (Fig. 3e).

**Figure 4:**
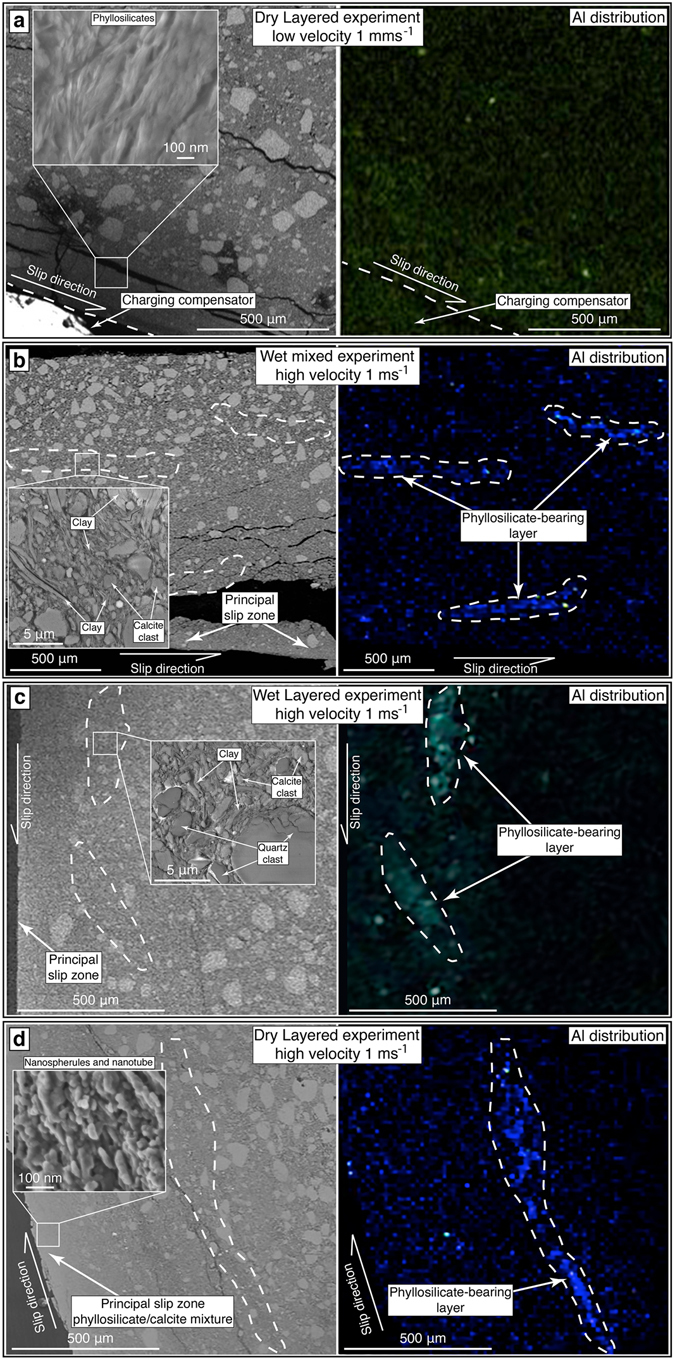
Experimental microstructures. **(a)** Left: FE-SEM image of dry layered gouge experimentally-sheared at subseismic velocity (0.001 ms^−1^). Grain size reduction occurs toward the principal slip zone. Inset shows a detail of phyllosilicate lamellae showing no nanospherules or nanotubes that occur, in contrast, in samples from experiments at seismic velocity (see **d**). Right: EDS map showing no concentration and segregation of phyllosilicates along distinct layers that occur, in contrast, in samples from experiments at seismic velocity (see **b–d**). FE-SEM image of **(b** left**)** wet mixed gouge experiment and **(c** left**)** wet layered gouge experiment sheared at seismic-slip velocity (1 ms^−1^) showing grain size reduction toward the principal slip zone. EDS map shows segregation and concentration of phyllosilicates along micrometer-thick layers both for mixed (**b** right) and for layered (**c** right) gouges under wet conditions. Insets show carbonate clasts within phyllosilicate-bearing layer wrapped by phyllosilicates. **(d)** Left: FE-SEM image of dry layered gouge experiment sheared at seismic-slip velocity (1 ms^−1^) showing, toward the principal slip surface, grain size reduction and compaction stronger than wet gouges. Inset shows the nanostructures from the principal slip zone. These structures consist of clumped and chained nanospherules and nanotubes identical to those observed within phyllosilicates along the Tre Monti principal fault (see Fig. 2e and f). Right: EDS map showing concentration and segregation of phyllosilicates along a micrometer-thick layer.

Using FE-SEM, we investigated the micro- and nano-structures obtained from the experimental gouges. Layered and mixed gouges sheared at subseismic velocity have a fabric consisting of randomly distributed particles of phyllosilicates and calcite (Fig. 4a). On the contrary, layered and mixed gouges sheared at high velocity (i.e., seismic slip rate, 1 ms^−1^), both in wet and in dry conditions, have similar microstructures characterized by (1) reduced grain size within a ~500 μm-thick deformation zone along the slip surface and by (2) phyllosilicate segregation and concentration along micrometer-thick layers (Fig. 4b,c and d). These layers were absent within the pre-sheared gouges (see Supplementary Fig. S3). Phyllosilicate-bearing layers form discontinuous patches rather than continuous layers and include carbonate clasts floating within a fine-grained matrix consisting of phyllosilicate lamellae wrapping around clasts (Fig. 4b and c). These microstructures are identical to those of natural micrometer-thick phyllosilicate-bearing layers observed within the Tre Monti Fault ultracataclasite (Fig. 2d).

Mixed and layered gouges in dry conditions show extreme grain size reduction and shear localization along a ~50 μm-thick principal slip zone (Fig. 4d). This principal slip zone is a mixture of phyllosilicates and calcite consisting of slip-parallel ~50 nm thick fibers composed by an array of nanospherules and nanotubes (Fig. 4d). In places, individual nanospherules clump together to form granular bulbous zones. These structures are very similar to phyllosilicate nanostructures observed within the Tre Monti Fault phyllosilicate-bearing layers (Fig. 2e and f). On the contrary, the principal slip zones of experiments conducted at subseismic slip rates are not characterized by nanospherules or nanotubes (Fig. 4a). Given the very slow creep rates and large cumulative slips possibly accommodated by natural slipping zones, we cannot rule out that clay nanospherules and nanotubes within the Tre Monti Fault (Fig. 2e and f) may have formed aseismically, as suggested by Verberne *et al*.^53^ for nanospherules within calcite gouges sheared at subseismic velocity (e.g., slip velocity 1 µm/s). However, in the case of the Tre Monti Fault, the coexistence of (1) clay nanospherules and nanotubes within phyllosilicate-bearing layers (Fig. 2e and f) similar to those observed within other phyllosilicates sheared at co-seismic slip-rates (compare with Fig. 9c in Ujiie *et al*., 2011), (2) truncated carbonate clasts (observed only within calcite gouges sheared at co-seismic slip rates^54^) along phyllosilicate-bearing layers (Fig. 2c), and (3) co-seismic displacements documented by *in-situ* ^36^Cl fault scarp exposure dating^30^, possibly suggests that phyllosilicate-bearing layers formed during fault slip at co-seismic slip rates. Moreover, we emphasize that no nanospherules and nanotubes were generated during low-velocity experiments both in dry and in wet conditions (Fig. 4a). Accordingly, we hypothesize that the Tre Monti Fault hosted co-seismic slip propagation along phyllosilicate-bearing layers up to shallow levels.

## Discussion and Conclusions

We conclude that calcite gouges with an illite/smectite content as low as 3% localized (i.e., due to clay smearing and/or pressure-solution/neoformation processes) along pre-existing micrometer layers (Fig. 3a) weaken faster than calcite and mixed calcite-phyllosilicate gouges (Fig. 3c,d, and e) at seismic slip rates (1 ms^−1^) both in dry and in wet conditions. Therefore, the occurrence of such phyllosilicate-bearing layers can facilitate fault weakening within carbonate-hosted faults, enhancing co-seismic slip propagation up to the Earth’s surface in continental domains. Clay-bearing layers can also be produced during co-seismic slip by clay segregation and localization from an initial mixture of clay and calcite, as shown within gouges sheared at seismic slip rates (1 ms^−1^; Fig. 4b–d). The segregation of clay into numerous phyllosilicate-bearing layers can act as a self-enhancing process, promoting further fault weakening during earthquakes along newly formed phyllosilicate-bearing layers. Regardless such clay concentration (Fig. 4b–d), mixed and layered gouges sheared at seismic slip rates, both in wet and dry conditions, have similar microstructures characterized by reduced grain size within a ~500 μm-thick deformation zone along the slip surface (Fig. 4b–d). This evidence suggests that the starting layered vs. mixed configurations have no influence on the final gouge microstructure. On the contrary, mechanical data show that layered gouges weaken faster than mixed gouges (Fig. 3c). This evidence suggests that the initial gouge setting (layered vs. mixed) exerts a strong mechanical control on the weakening behaviour of the gouges regardless of the final microstructures.

The steady-state friction coefficient measured in our experiments (~0.4) is somewhat higher than that (~0.2) resulting from previous experiments performed on carbonate gouges at co-seismic slip rates^13, 54^. This difference is likely due to the lower applied normal stress (i.e., 8.5 MPa and 5 MPa in dry and wet conditions, respectively) set in our experiments to simulate co-seismic slip at depth <500 m. Moreover, we used a metal gouge holder hardened with WC (i.e., tungsten carbide), which owns a thermal diffusivity of 3.5 10^−5^ m^2^s^−1^ about ten times larger than that of calcite gouges and natural rocks^55^. The metal holder allowed easy diffusion of heat, which buffered the temperature rise during shearing of the gouge^56^. Since most co-seismic weakening mechanisms are temperature-dependent^9^, the thermal properties of the sample holder influence strain localization, heat production, temperature rise and the measured friction coefficient (i.e., higher the temperature in the slipping zone, lower the friction coefficient)^56^. Similarly, regarding the extrapolation of our experimental measurements of the friction coefficient to natural conditions, the lower thermal diffusivity of rocks and the higher normal stress at depth can further reduce (i.e., below 0.4) the friction coefficient during earthquakes^9, 10, 57^.

In summary, we propose that the 3 wt.% of clay concentrated within pre-existing ultra-thin layers could be the lower boundary for facilitating dynamic weakening during earthquake slip at shallow crustal levels in continental domains. The dynamic weakening effect can be even stronger if the phyllosilicate content exceeds 3 wt.%^22^.

## Methods

### Description of starting materials

We used natural materials collected in the field from the foliated carbonate cataclasite and the phyllosilicate-rich deposits to perform high velocity friction experiments (Fig. 2a). The foliated cataclasite consists of 100% calcite whereas the total phyllosilicate content of the phyllosilicate-rich deposits is ~30% (i.e., the phyllosilicate rich powder used for the experiments contain ~30% of phyllosilicates; Table S2). We used 4.5 g of gouge for each experiments resulting in ring-shaped gouge layers (35/55 mm int./ext. diameters), with initial thicknesses of ~3 mm (Fig. 3a).

### Description of XRD analysis performed on natural rocks

We performed powder X-ray diffraction (XRPD) analyses and energy dispersive spectroscopy (EDS) to determine the mineralogy of natural fault rocks and experiment materials. In particular, we performed XRPD analyses on the insoluble residue from the ultracataclasite and from the natural phyllosilicate-bearing layers. We isolated the insoluble residues using HCl acid to dissolve calcite. XRPD data were obtained using a PANalytical θ-θ diffractometer equipped with a long fine-focus Cu X-ray tube (operating at 40 kV and 40 mA) and a real-time multiple strip (RTMS) detector (X’Celerator). The scan was performed over the 2θ range of 3–80°, with a virtual 2θ step size of 0.017°, and a counting time of 100 s/step. The program High Score Plus (PANalytical) was used for phase identification and quantitative phase analysis with Rietveld refinement^58^.

### Descriptions of experimental rotary shear apparatus and gouge holder

We performed friction experiments on the Slow to High Velocity rotary shear Apparatus (SHIVA) at INGV, Roma^59^. The apparatus has a horizontal setup, with the rotary column on one end, the sample chamber in the middle, the axial column and loading system side at the other end. Rotary motion is supplied to the sample pair by two brushless engines of different size. The larger engine operates in a velocity range of 1–3,000 RPM, and owns a peak torque of 932 Nm and a nominal maximum power output of 280 kW. We performed experiments using a dedicated metal sample holder (Fig. 3a) appropriately designed for shearing gouges at high velocities^13^. Ring-shaped layers of gouge (55 mm external diameter, 35 mm internal diameter) are confined by outer and inner rings of tungsten carbide that rotate and slide over a base disc of the same material. The base disc and the rotary base plate are etched with a cross-hatch pattern to provide roughness at the boundaries of the gouge layer. The normal load, exerted by an air actuator, was applied on the gouge layer by the stationary column of SHIVA. Normal load applied to the outer and inner rings is independent of that applied to the gouge layer, and is modulated by five outer springs and one inner spring: normal load is proportional to the spring stiffness and to the amount of compression during each experiment. Given that we used the same layer thickness for each experiment, we consider the normal load on the outer and inner rings to be the same for each experiment. Calibration of the gouge holder is discussed in detail in Smith *et al*. (2013).

### Description of experimental conditions

We conducted dry experiments at room humidity conditions, whereas, in water-saturated experiments, we water damped gouges with 0.5 ml of distilled water. Gouge materials for tests were oven-dried at 40 °C for days before the experiments. Experimental slip rates were set at 0.001 ms^−1^ and 1 ms^−1^, for a total sliding displacement of 0.5 meter under water-saturated (wet) and dry conditions. We held normal stress constant at 8.5 MPa and 5 MPa for dry and wet experiments, respectively. The SHIVA apparatus does not allow the measurement/control of pore pressure during the wet experiments. Accordingly the measured friction should be considered as apparent (or effective), as suggested by Chen *et al*. (2017), for the unknown pore pressure within the gouge. The experimental sequence progressed as follows: gouges were initially pre-sheared at 1 cms^−1^ for 10 cm of total slip under 0.5 MPa of normal load, both in low velocity and in high velocity experiments and both in dry and in wet conditions. In all experiments, gouges were initially pre-sheared at 0.01 ms^−1^ for 10 cm of total slip under 0.5 MPa of normal load. This procedure was done to properly simulate the behaviour of our natural fault rocks (Fig. 2b), which were (pre-) sheared by multiple slip events and to limit the pressurization effects due to gouge compaction, as also done in previous experiments in rotary shear apparatuses^50^. After, gouges were loaded to the target normal stress and were sheared up to the target displacement. For high velocity experiments, acceleration and deceleration rates were of 24 ms^−2^. After the experiments, gouges were cohesive and flinty. We impregnated gouge fragments under vacuum using a LR-white resin. We then prepared petrographic sections from the impregnated gouges cut perpendicular to the gouge layers and approximately parallel to the slip direction. We studied micro- and nano-structures (both natural and experimental) through scanning electron microscope on polished thin sections ~30 µm thick. All samples were uniformly coated with 1–2 nm thick chrome particles. We used three different facilities. (1) A thermal FE-SEM Zeiss Merlin Gemini 2 with 0.7 nm of resolution and four detectors (two in lens detector for high resolutions) at CERTEMA (Multidisciplinary technology laboratory, Grosseto, Italy). This device is equipped with a high sensitivity EDS detector (active area of 50 mmq) and with a 5 crystals WDS spectrometer. (2) A thermal FE-SEM JEOL (jsm6500f) at INGV (National Institute of Geophysics and Volcanology), Rome, with 2.5 nm of resolution and two detectors. EDS has an active area of 25 mmq. (3) A FE-SEM Zeiss Auriga at CNIS (Center for Nanotechnologies Applied to Engineering, Sapienza University of Rome, Italy) with 1 nm of resolution and three detectors (one in lens detector for high resolutions). All thin-sections were examined under optical microscope at various magnifications prior than SEM analyses.

## Electronic supplementary material

Supplementary informations for Ultra-thin clay layers facilitate seismic slip in carbonate faults
