# Supplementary material for: Ultra-thin clay layers facilitate seismic slip in carbonate faults
Source: Sci Rep. 2017 Apr 6;7:664. doi: 10.1038/s41598-017-00717-4 (PMC5429680; doi:10.1038/s41598-017-00717-4)
Supplement: Supplementary file 1 — Supplementary informations for Ultra-thin clay layers facilitate seismic slip in carbonate faults [file 41598_2017_717_MOESM1_ESM.pdf]

Ultra-thin clay layers facilitate seismic slip in carbonate faults

Luca Smeraglia[1], Andrea Billi[2], Eugenio Carminati[1,2], Andrea Cavallo[3], Giulio Di Toro[4,5,6], Elena Spagnuolo[5], and Federico Zorzi[6]

[1]Dipartimento di Scienze della Terra, Sapienza University of Rome, Italy.

[2]Consiglio delle Nazionale Ricerche, IGAG, Rome, Italy

[3]CERTEMA, Multidisciplinary technology laboratory, Cinigiano, Grosseto, Italy

[4]School of Earth, Atmospheric and Environmental Sciences, The University of Manchester, United Kingdom

[5]INGV, Istituto Nazionale di Geofisica e Vulcanologia, Rome, Italy

[6]Dipartimento di Geoscienze, Padova University, Italy

Luca Smeraglia*(corresponding author): luca.smeraglia@uniroma1.it

Andrea Billi: andrea.billi@cnr.it

Eugenio Carminati: eugenio.carminati@uniroma1.it

Andrea Cavallo: a.cavallo@laboratoriotecnologicogrosseto.it

Giulio Di Toro: giulio.ditoro@manchester.ac.uk

Elena Spagnuolo: elena.spagnuolo@ingv.it

Federico Zorzi: federico.zorzi@unipd.it

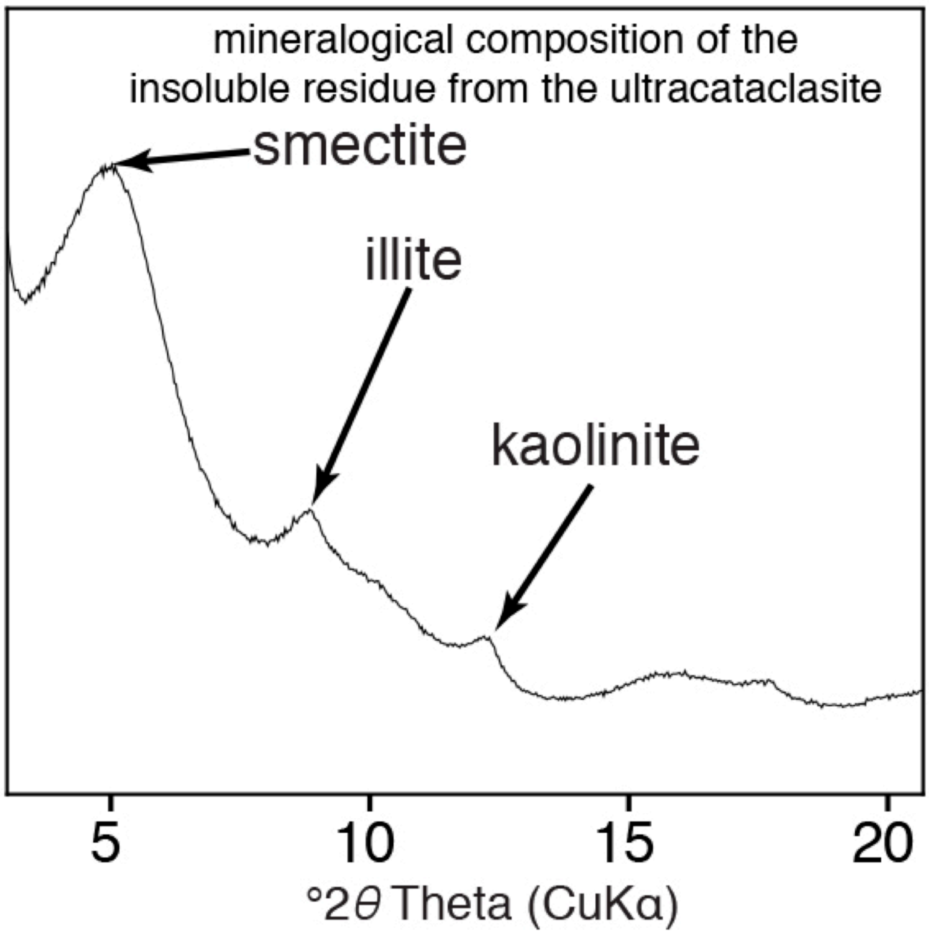

Supplementary Fig. S1. Powder X-ray diffraction (XRPD) traces of the insoluble residue from the ultracataclasite Diffraction peaks showing the occurrence illite, smectite and kaolinite within the insoluble residue (~1.5% wt.) from the ultracataclasite.

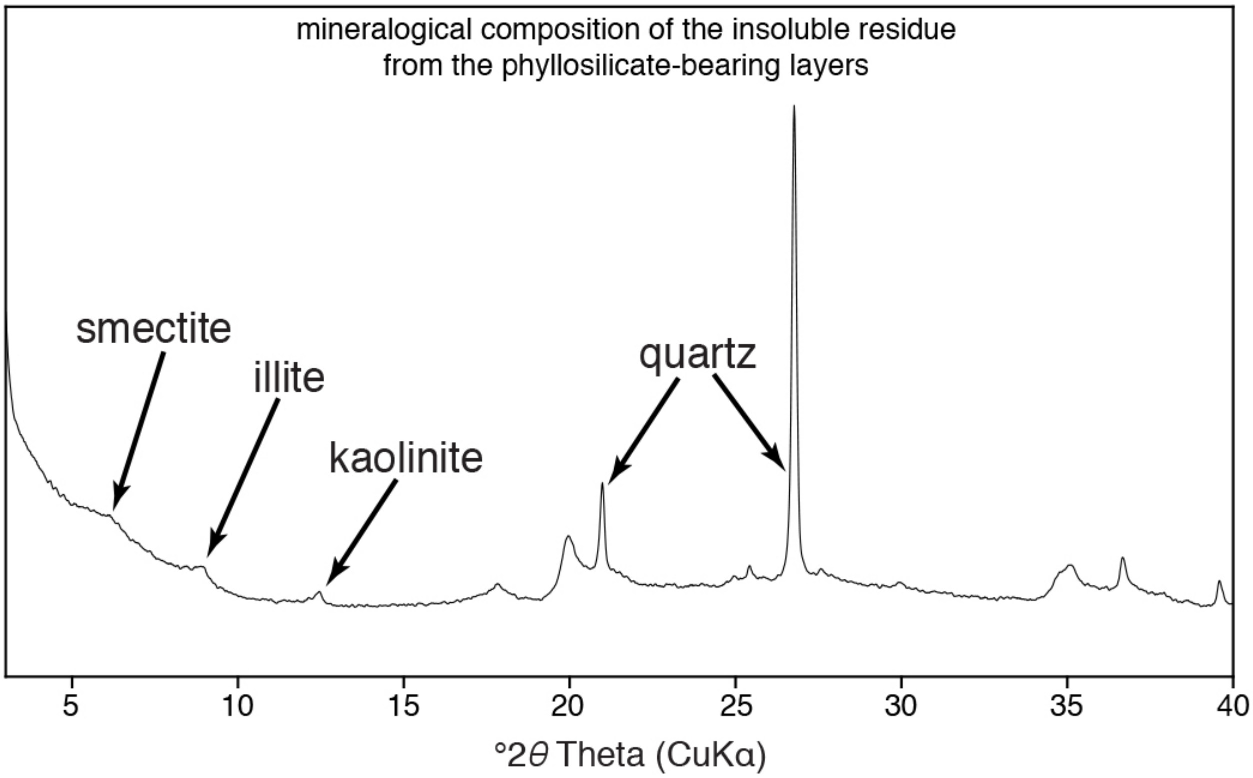

Supplementary Fig. S2. Powder X-ray diffraction (XRPD) traces of the insoluble residue from the phyllosilicate-bearing layer within the ultracataclasite. Diffraction peaks showing the occurrence illite, smectite, kaolinite, and quartz within the insoluble residue (~8.6% wt.) from the phyllosilicate-bearing layer within the ultracataclasite.

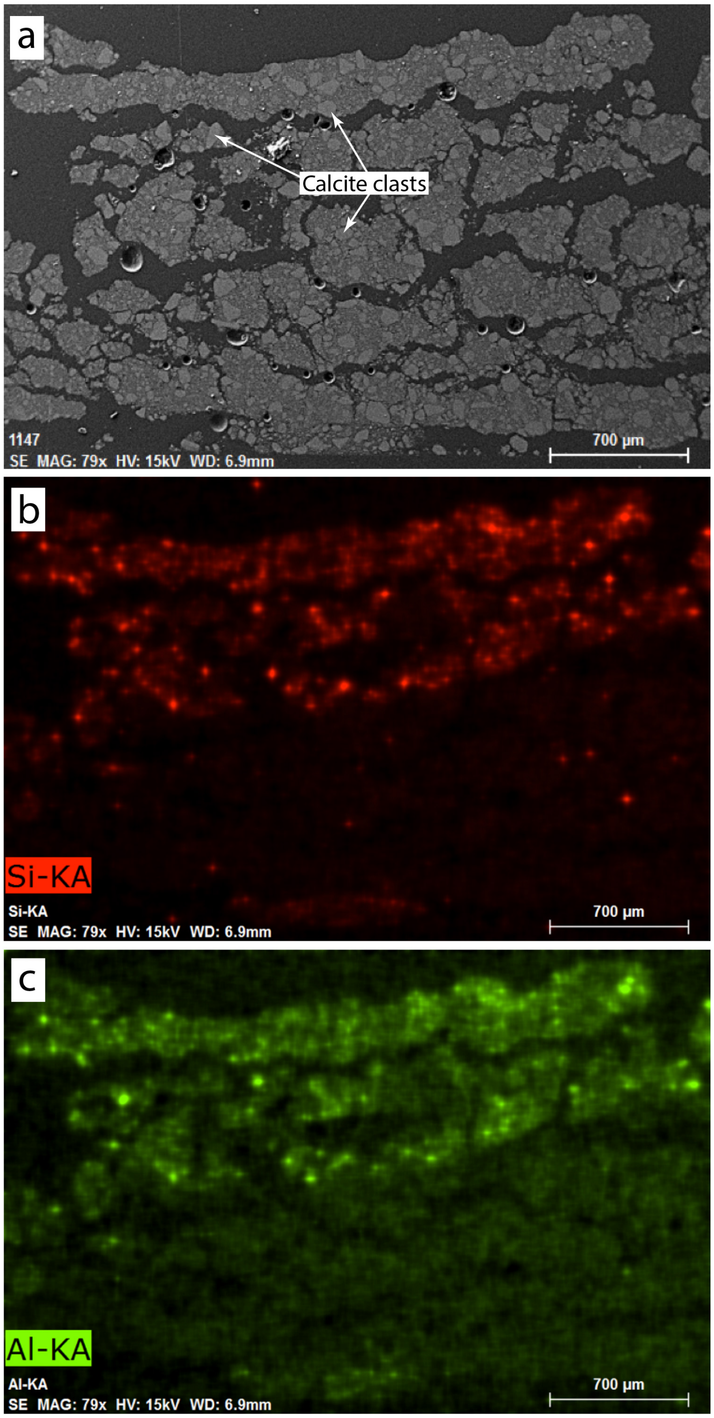

Supplementary Fig. S3. Pre-shear configuration of dry layered experiment. (a) FE-SEM image showing microstructures of pre-shear configuration of dry layered experiment. EDS maps showing any silica **(b)** and aluminum **(c)** concentration along the phyllosilicate-rich layer. Silica and aluminum are indicative of the presence of phyllosilicate.

Location	Earthquake	Date	Slip Type	Magnitude	Surface rupture length (km)	Surface displacement (m)
Greece	Sparta	–	N	–	20	3.50
Turkey	Menderes	22/02/1653	N	7.1	70	3.00
Iran	Vostiza	26/12/1861	N	6.6	13	2.20
Greece	Fokis	01/08/1870	N	6.7	6+	2.00
Greece	Civril	03/05/1875	N	6.5	10	1.10
Turkey	Emiralan	29/07/1880	N	6.5	10	0.40
Turkey	Banaz	30/09/1887	N	6.3	10	0.50
Turkey	Martin	27/04/1894	N	6.9	40	1.00
Greece	Mender	20/09/1899	N	6.9	40	1.00
Turkey	Struma	4/4/1904	N	7.2	25	2.00
Bulgaria	Scutari	1/6/1905	N	6.3	10	1.00
Albania	Marmara	9/8/1912	NR	7.4	50	3.00
Turkey	Burdur	3/10/1914	NR	7.0	23	1.50
Italy	Avezzano	13/1/1915	N	7.0	20	2.0
USA	Pleasant Valley	3/10/1915	N	7.6	62	5.8
Kenya	Laikipia	1/6/1928	N	7.0	31	3.3
Turkey	Plovdiv	14/4/1928	N	6.8	–	0.50
Bulgaria	Papazili	18/4/1928	N	6.9	50	3.5
Iran	Ieriss	26/9/1932	N	6.9	15	1.80
Greece	Saphane	25/6/1944	NR	6.0	18	0.30
Peru	Ancash	10/11/1946	N	7.2	21	2.1
USA	Fort Sage Mtns.	14/12/1950	N	5.6	9.2	0.20
Turkey	Sofades	30/4/1954	N	6.7	30	0.90
USA	Rainbow Mountain	6/7/1954	N	6.3	18	0.31
USA	Stiliwater	24/8/1954	N	6.9	34	0.76
Greece	Velestin	8/3/1957	NL	6.6	1	0.20
USA	Hebgen Lake	18/8/1959	N	7.6	26.5	6.1
Greece	Manyas	16/10/1964	NR	6.8	40	0.10
Turkey	Megalop.	1/9/1966	N	5.6	2	0.05
Greece	Acarnania	29/10/1966	N	5.8	4	0.40
Greece	Debar	30/11/1967	NL	6.6	10	0.50
Turkey	Alasehir Valley	28/3/1969	N	6.5	32	0.82
Turkey	Gediz	28/3/1970	N	7.1	41	2.8
Turkey	Burdur	12/5/1971	N	6.2	4	0.30
USA	Oroville	1/8/1975	N-RL	5.6	3.8	0.06
Greece	Thessaloniki	20/6/1978	N	6.4	19.4	0.22
Turkey	Volvi	20/6/1978	N	6.4	32	0.20
Greece	Almyros	9/7/1980	N	6.4	5.3	0.2
Italy	South Apennines	23/11/1980	N	6.9	38	1.15
Greece	Corinth	25/2/1981	N	6.4	19	1.5
Greece	Corinth	4/3/1981	N	6.4	13	1.1
Yemen	Dhamer	13/12/1982	N	6.0	15	0.03
USA	Borah Peak	28/10/1983	N-LL	7.3	34	2.7
Peru	Cuzco	5/4/1986	N	4.6	2.5	0.1
Greece	Kalamata	13/9/1986	N	5.8	15	0.18
New Zealand	Edgecumbe	2/3/1987	N	6.6	18	2.90
USA	Eureka Valley	17/5/1993	N	5.8	4.4	0.02
Greece	Kozani	13/5/1995	N	6.5	15	0.05
Turkey	Dinar	1/10/1995	NR	6.2	10	0.30
Italy	L'Aquila	7/4/2009	N	5.5	6	0.30
Mexico	El Mayor-Cucapah	4/4/2010	N	6.88	–	0.30

Supplementary Table S1. Non-exhaustive list of onshore extensional fault earthquakes, which caused surface displacement. Historical and instrumental extensional fault earthquakes that generated surface faulting up to about six meters of vertical displacement in different regions worldwide (e.g., Wells and Coppersmith, 1994; Ambraseys and Jackson, 1998; Martino et al.,

2014). *E = extensional; RL = right lateral; LL = left lateral. For earthquakes with an oblique component of slip, the subordinate sense of slip is listed after the primary slip type.

Lithology	Quartz (%)	K-feldspar (%)	Plagioclase (%)	Dolomite (%)	Calcite (%)	Clay minerals (%)
Ultracataclasite	0	0	0	0	98.5	1.5
Phyllosilicate-rich layers (ultracataclasite)	3	0	0	0	92	5
Cataclasite	0	0	0	0	100	0
Syn-orogenic deposit (Hemipelagic marls)	26	0	0	5	24	45
Syn-orogenic deposit (Siliciclastic sandstones)	31	3	13	11	12	30

Supplementary Table S2. Powder X-ray diffraction (XRPD) mineralogical composition of natural fault rocks and tested materials. The ultracataclasite consists of calcite (98.5% wt.) and clay minerals (1.5% wt.). Phyllosilicate-bearing layers consist of calcite (93% wt.), quartz (2% wt.), and clay minerals (5% wt.). The foliated cataclasite consists solely of calcite (100% wt.). Siliciclastic sandstones (Syn-orogenic deposits) consist of quartz (24%), k-feldspar (3%), plagioclase (11%), dolomite (11%), calcite (16%), pyrite (1%), muscovite/illite (21%), chlorite (9%), serpentine (3%), and non quantifiable low amount of smectite and kaolinite. Hemipelagic marls (Syn-orogenic deposits) consist of of quartz (16%), plagioclase (5%), dolomite (3%), calcite (32%), Mg-calcite (21%), muscovite/illite (12%), chlorite (4%), kaolinite (3%), serpentine (4%), and non quantifiable low amount of smectite.

wt%	Phyllosilicates	Detrital Mica	Plagioclase (albite)	K-feldspar	Quartz
Na_2O	0.4	0.7	11.6	0.9	0.1
MgO	3.9	5.4	0.0	0.3	0.2
Al_2O_3	23.4	29.1	19.0	17.3	0.8
SiO_2	55.0	48.8	68.6	69.3	96.1
K_2O	2.7	3.6	0.0	11.8	0.2
CaO	5.0	5.8	0.8	0.0	0.6
TiO_2	1.4	1.1	0.0	0.4	0.3
MnO	0.7	1.6	0.0	0.0	1.3
Fe_2O_3	6.4	2.3	0.0	0.0	0.5
sum	99.0	98.4	100.0	100.0	100.0

Supplementary Table S3. Average EDS composition of the non-carbonate minerals within the ultracataclasite. Results show the occurrence of phyllosilicates, detrictal mica (mainly illite/muscovite), plagioclase, K-feldspar, and quartz. The chemical data suggest that phyllosilicates and detrital mica are an assemblage of muscovite, illite, chlorite, and smectite.

Number of experiment	Type of experiment	Velocity (m/s)	Normal stress (MPa)	Distance to the onset of dynamic weakening (m)	Steady state friction	Fracture energy (MJ/m^2)	Total displacement (m)
1146	Dry Layered	1	8.5	0.17	0.40	0.461	0.5
1147	Dry Random	1	8.5	0.26	0.48	0.473	0.5
1155	Dry Carbonate	1	8.5	0.25	0.45	0.570	0.5
1150	Wet Layered	1	5	0.14	0.35	0.217	0.5
1149	Wet Random	1	5	0.18	0.34	0.232	0.5
1156	Wet Carbonate	1	5	0.21	0.36	0.262	0.5
1273	Dry Layered	1	8.5	0.16	0.33	0.461	0.5
1272	Dry Random	1	8.5	0.20	0.35	0.589	0.5
1271	Dry Carbonate	1	8.5	0.22	0.40	0.618	0.5
1276	Wet Layered	1	5	0.009	0.27	0.112	0.5
1275	Wet Random	1	5	0.02	0.29	0.193	0.5
1274	Wet Carbonate	1	5	0.1	0.41	0.386	0.5

Supplementary Table S4. Summary of experimental conditions and mechanical results of friction experiments.
